# Nanoparticles in explosives detection – the state-of-the-art and future directions

**DOI:** 10.1007/s12024-017-9903-4

**Published:** 2017-08-12

**Authors:** William J. Peveler, Sultan Ben Jaber, Ivan P. Parkin

**Affiliations:** Department of Chemistry, University College London, 20 Gordon St, WC1H 0AJ, London, UK

## Introduction

Nanoparticle-ligand systems can be targeted to specific analytes to effect a change in the properties of the nanoparticles. We will examine two examples in which the electromagnetic properties of the nanoparticles (arising from their small size) are altered by analyte binding, and can be applied as a transducer in a chemical sensing system for explosive analytes. The first property reviewed is the surface plasmon resonance (SPR) band of colloidal gold nanoparticles (AuNPs), and the second, the fluorescence of colloidal semiconductors (quantum dots – QDs).

Sensors based on these nanoparticle properties have the potential to detect picomolar or lower concentrations of explosive analytes, and can operate for both solution phase and gas phase detection [1–3]. In addition, measurement of the signal produced by the nanoparticle transducer uses standard scientific instrumentation, making it easier to build complete detection systems from standard components - an important consideration [4].

## Example 1 - gold nanoparticles

In AuNPs the free electrons of the metal surface interact strongly with light causing large oscillations in the surface electromagnetic field. The particles therefore absorb light strongly at the particular resonant frequencies of these electrons, giving rise to SPR bands.

**Fig. 1:**
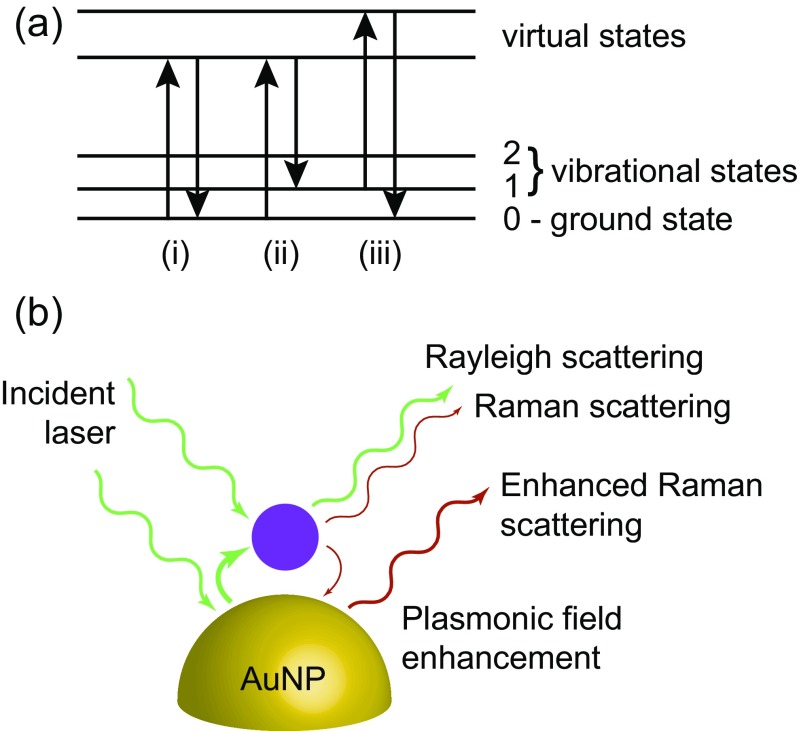
**a** Basic inelastic scattering modes that give rise to Raman spectra. (i) Rayleigh scattering - elastic scattering that is discarded from the spectrum. (ii) Stokes Raman scattering, where the change in wavelength of the emitted photon gives the Raman signal. (iii) Anti-Stokes Raman scattering - much weaker than the Stokes equivalent. **b** Electromagnetic enhancement of Raman scattering for a physisorbed analyte on an AuNP surface. Both incident laser light and outgoing Raman light are enhanced by the particle plasmonic field. Adapted from Guerrini & Graham [9]

One method to exploit the plasmons of AuNPs for sensing is to use them in surface enhanced Raman spectroscopy (SERS). A Raman spectrum is a powerful way to fingerprint a molecule, using incident light to excite Raman active vibrational modes (Fig. 1a), causing inelastic scattering of the photons, and giving rise to a unique spectrum that provides information on molecular shape and connectivity. The spectrum obtained from an unknown analyte can be compared to a library of known spectra and used to identify a threat. However, Raman scattering is very weak, and so detection of low levels of analyte, enhancement is required.

If a molecule is bound (chemi- or physisorbed) to a metal surface, incident light (usually a monochromated laser pulse) excites the surface plasmons, inducing polarization in the bound molecules, increasing the amount of inelastic scattered light from the Raman vibrational modes (Fig. 1b). This leads to a signal enhancement of up to E^4^, where E is the electric-field magnitude. The intensity of the SERS effect is largely attributed to the monolayer of molecules absorbed to the nanoparticles, and is highly dependent on the form (and hence plasmonic field) of the nanoparticles [5–8]. This adsorption onto the substrate also creates new vibrational selection rules, and surface-complex formation can lead to altered electronic properties of the absorbed molecule [9].

The best SERS enhancement is achieved by having strong localized plasmons, that fall within the wavelength of the Raman laser excitation. For this reason gold and silver are often chosen, as their SPR bands are typically within 400–800 nm, which is easy to access with a visible laser [10]. In addition they are chemically inert and thus stable against air, and strong oxidizing or reducing agents. Other materials have been successfully applied as SERS substrates, such as other noble metals (Pt and Pd) and even transition metals and semiconductors [11, 12].

Strong plasmon hotspots created in between individual particles can improve the SERS enhancement effect. This can be achieved through aggregation of the particles, either in solution by trapping at an interface or chemical aggregation with linking molecules, or by solvent removal [13–15].

In recent years SERS on nanoparticulate colloids has been heavily applied to the detection of illicit materials, such as explosives [16, 17]. The technique has been shown to be capable of detecting a range of high explosives, with good detection limits (into the nanomolar region or below), even with raw colloidal solutions of Au and AgNPs, such as those applied by the group of Hernández-Rivera [18, 19]. By aggregating AuNPs at an interface, Edel et al. created a regular monolayer array, which showed enhanced sensitivity to a range of compounds, including some explosives [20].

A more targeted approach has been taken by utilizing NPs functionalized with cysteine to form Mesienheimer complexes with nitroaromatics [21, 22]. Xu et al. demonstrated enhanced detection of DNT by using cyclodextrin coated triangular nanoprisms of gold [23]. SERS also extends beyond the nitroaromatics, for example it has been shown that RDX can be detected at concentrations as low as 0.15 mg/L in ground water samples [24]. This illustrates a key benefit of SERS, that it is label free, requiring no special binding groups to target particular analytes, but that it can become more targeted with designed substrates.

SERS is a very useful technique for detection of explosives as it can can detect solution or vapor phase materials at very low concentrations. The individual Raman fingerprint of each different molecule makes specificity high, however in complex mixtures, deconvolution can be a challenge [25]. A second problem is the dependence of the Raman signal on the SERS substrate. Particular vibrational modes in the Raman spectrum can be enhanced or suppressed depending on the binding mode of the analyte, and if the substrate (e.g. AuNP concentration and aggregation amount) is not identical in every instance then the fingerprints may differ slightly, causing loss of specificity. Therefore, a key requirement is the development of cheap, identical SERS substrates, that exhibit powerful enhancement, ensuring strong and repeatable spectral fingerprints can be obtained on each use.

**Fig. 2:**
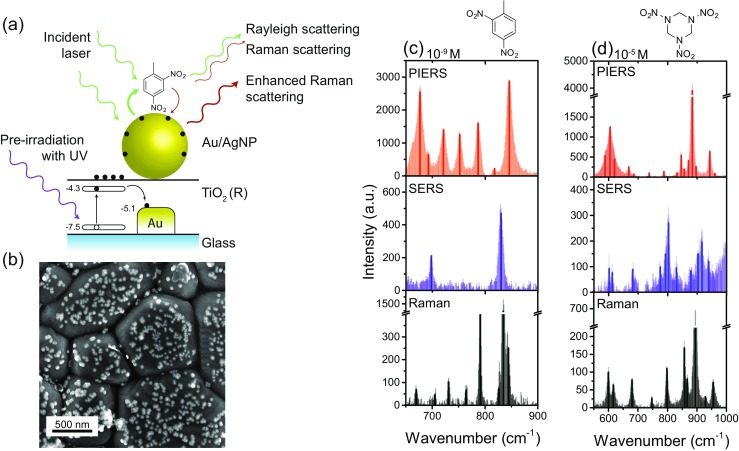
Illustration of (**a**) the proposed PIERS mechanism, (**b**) SEM of a PIERS substrate and (**c,d**) exemplar sensing results for DNT and RDX. Adapted from [2]

Recently we reported a new method of designing such substrates based on a new enhanced Raman technique – photoinduced enhanced Raman spectroscopy – PIERS. It was shown that by combining semiconducting TiO_2_ substrates with plasmonic AuNPs, and pre-irradiating the material, a PIERS substrate was created that displayed an order of magnitude enhancement over conventional SERS techniques [2]. We performed sensing of DNT, TNT, RDX and PETN explosives in solution, with high quality spectral finger prints obtained even at sub-nanomolar concentrations (Fig. 2). In particular nanomolar concentrations of DNT and TNT were detected both in solution and in the vapor phase, demonstrating that this PIERS technique might have interest for stand-off detection of explosives. We also showed that via the pre-irradiation step the substrates could be fully cleaned over several cycles, meaning the same substrate can be used multiple times, unlike many commercial SERS substrates on the market today.

We undertook a thorough investigation into the mechanism of this enhancement and suggest that it arises from interaction between the irradiated TiO_2_ and gold nanoparticles, causing improved charge transfer and electromagnetic enhancement at the surface of the substrate (Fig. 2). Further investigations into the underlying mechanism and optimization for field detection of explosives, and other threats, are ongoing.

## Example 2 - quantum dots

**Fig. 3:**
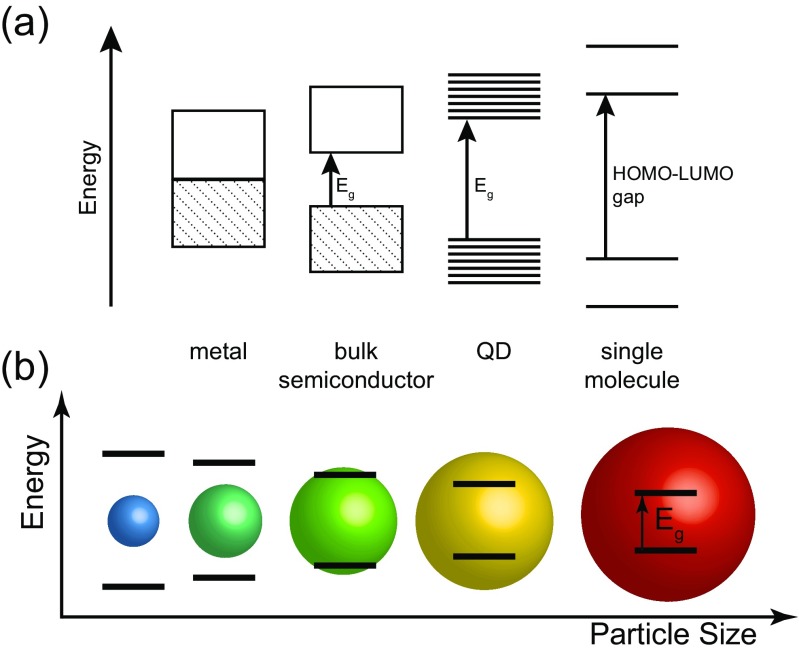
**a** Schematic of band structures of metals, semiconductors, quantum dots (QD) and single molecules, showing the increase in band gap (E_g_) as confinement is increased. At the single molecule scale the energy bands become completely discrete, and the QD sits between this system and the band model of semiconductors. **b** Graphic illustrating the change in QD band gap and photoluminescence emission wavelength (colour) with increasing particle size

Quantum dots (QDs) are semiconducting nanoparticles which are small enough to confine a generated hole-electron pair (exciton) within all 3 spatial dimensions, leading to quantization of the energy levels. This causes the electronic structure of the material to sit between a classical semiconductor, and a molecular material (Fig. 3a).

The result of this quantization of states is that the nanoparticles display sharp photon absorption and emission bands, and the band gap is closely related to the size of the nanoparticle. The fluorescence arises from photoexcitation of the nanoparticles, causing exciton formation. Recombination of this exciton will then occur through radiative (fluorescence) or non-radiative (trap-states, oxidation, energy transfer) pathways. The fluorescence from QDs is easily tuned to the visible or near-IR region of the spectrum, by choice of semiconductor material and particle size, making QDs useful fluorophores (Fig. 3b).

Introducing molecules around the surface of the QD will affect the rate of recombination of the exciton, and may also disrupt its recombination. In particular, the conduction band electron may be lost to a local species in a process termed photoinduced electron transfer (PET). In PET the loss of the excited electron to the acceptor results in the prevention of recombination, and thus the loss of fluorescence. The more efficient this process, the larger the fluorescence quenching of the system.

This electron transfer mechanism has been used extensively to transduce the presence of analytes in proximity to a QD, and thus form the sensor element in a chemical sensor [26–28]. The surface of the QD can be targeted to certain analytes by the placement of receptors on the surface that preferentially bind the analyte and bring it into close proximity with the QD, facilitating the PET mechanism, and quenching the observed fluorescence [29].

QDs, therefore, have many properties of interest to chemical sensing - they exhibit high fluorescence quantum yields, are resistant to photobleaching, and have broad absorption giving rise to narrow emission bands. This means they lend themselves well to a multiplexed or multichannel fluorophore system, with a single excitation wavelength causing emission from many different species of varying color. The surface of the particles is easily modified with targeting ligands to allow specific and sensitive fluorescence enhancement or quenching on association with an analyte via a PET mechanism.

In the security domain, QDs have been used for approximately 10 years as a fledgling sensor for explosives. Due to explosives’ (particularly nitro/conventional explosives’) electron deficient nature, they make excellent PET quenchers [30]. Initially antibody targeting of explosives was used, but this is complicated by the procedures to obtain, purify and conjugate the antibodies [31, 32]. A cheaper and simpler targeting approach has been the formation of Meisenheimer complexes, using surface amines to bind nitroaromatics, such as TNT or picric acid [33–38]. This system can be highly specific towards nitroaromatic materials, but is very difficult to apply more widely to other explosives (even DNT). A novel donor/acceptor system for the sensing of explosives beyond TNT has been pioneered by Willner et al. They have utilized both a redox couple based on NAD^+^/NADH and a donor/acceptor scheme, based on surface bound dopamine derivatives, to sense a range of explosives, including RDX [39, 40].

To further this work we have recently exploited the attractive optical properties of QDs to build an explosive sensing array of quantum dots. By combining multiplexed fluorophores with variable response do different explosives, with array statistics, it was possible to ‘fingerprint’ 5 different explosives and identify them at low concentration [1]. This has potential applications in waste-water management and testing, as well as drinking water evaluation in areas where explosives contamination is a health issue, such as ordinance ranges and manufacturing sites, as well as for safety management.

**Fig. 4:**
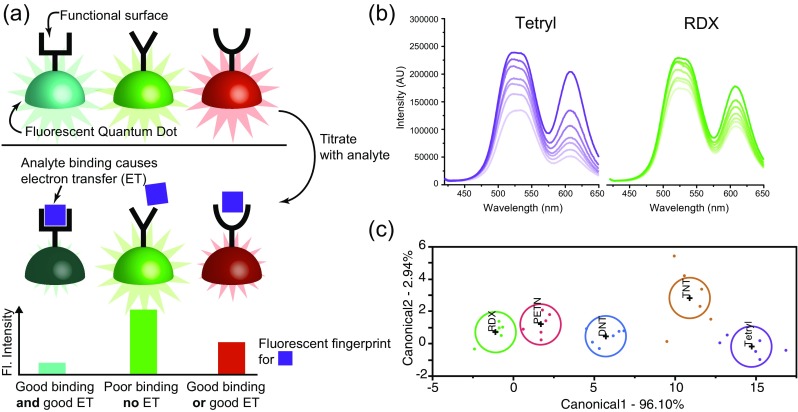
Array based sensing of explosives. (**a**) Schematic of a QD array for PET based sensing of explosives. (**b**) Fluorescent quenching by two explosives. (**c**) Statistical discrimination of the fluorescent fingerprints for different explosives. Adapted from [1]

In this system, the surfaces of the QDs were modified with a range of supramolecular functionalities to control their selective interactions with different explosives, and DNT, TNT, RDX, PETN and tetryl were successfully detected and discriminated using the array of just three QDs. It was also shown that the QDs in the array could operate in the format of a paper-based test, in addition to the solution-based assay. Limits of detection down to ppb levels were obtained, and most importantly the array could read out information on what type of explosive was present, rather than just if there was a particular single explosive or not (Fig. 4).

## The future?

Nanomaterials have shown great promise in the explosives detection field and it is likely that future commercial developments in this area will make some use of these types of matter. Two key areas of interest are the Raman enhancing properties of plasmonic gold nanoparticles, and the fluorescent nanomaterials such as quantum dots that can be used in complex photonic systems for sensing different types of explosive at low levels. Each of these has shown their worth in the laboratory and efforts must now focus on more rigorous device design and field testing to move towards end-user applications.
